# "Hot and Achy": A Case of an Extensive Spinal Epidural Abscess

**DOI:** 10.7759/cureus.60876

**Published:** 2024-05-22

**Authors:** Vinita D Yadav, Devi Parvathy Jyothi Ramachandran Nair, Shefali Amin, Manish Shrestha, Pavani Pagolu

**Affiliations:** 1 Internal Medicine, Reading Hospital/Tower Health, West Reading, USA

**Keywords:** internal medicine, neurology, fevers, back pain, spinal epidural abscess

## Abstract

We present a case of a 94-year-old female who presented to the emergency room with a fever and generalized weakness without an initial obvious source of infection. Throughout admission, she continued to be febrile despite broad-spectrum antibiotics. Several days into admission, the patient complained of severe back pain, necessitating magnetic resonance imaging (MRI) of the entire spine. The imaging revealed an extensive epidural fluid collection consistent with a spinal epidural abscess. Fortunately, she did not have any neurological deficits and was treated conservatively with IV antibiotics with improvement. This case highlights this rare presentation and the importance of early diagnosis and management of spinal epidural abscesses.

## Introduction

Spinal epidural abscess (SEA) is an uncommon, life-threatening infection of the space between the spinal dura and vertebral periosteum. It is typically located in the thoracolumbar region, as it consists of a large epidural space with an abundance of infection-prone fat, arteries, and veins; however, it may eventually span multiple levels. Common sites of origin include skin and soft tissue infections, complications of spinal surgery, or other invasive procedures involving the spine. It may also occur due to hematogenous spread. Patients may present with back pain, fever, and neurological deficits, however, this triad is only experienced in 8-15% of cases; therefore, prompt diagnosis is often missed. More often than not, rapid progression to late-stage disease with paralysis occurs in 34% of cases [1,2]. This can be seen with extensive disease defined as the involvement of more than five vertebral levels [1]. Remarkably, the case described in this report presents a rare instance of a patient with minimal symptoms who was found to have extensive SEA spanning nearly the entire spine.

## Case presentation

**Figure 1:**
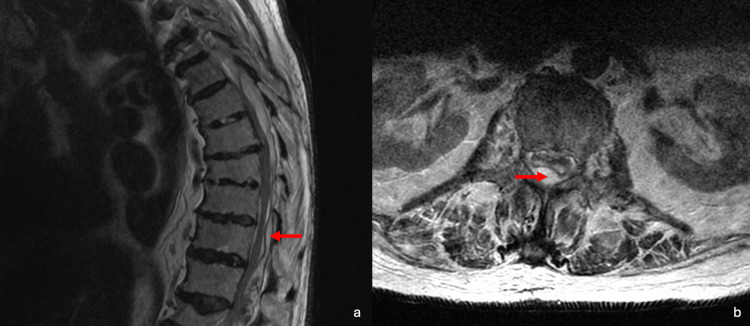
Epidural abscess (red arrows) found in the thoracic spine (a) and the lumbar spine (b)

We present the case of a 94-year-old female with a past medical history of coronary artery disease status post remote stent placement on dual-antiplatelet therapy with aspirin and clopidogrel, bilateral internal carotid artery stenosis, moderate aortic stenosis, hiatal hernia, gastroesophageal reflux disease, diarrhea-predominant irritable bowel syndrome, hyperlipidemia, and hypertension. She presented to the hospital with concerns of fever and generalized weakness for the past week. She could not ambulate or get out of bed, which was unusual for her. At baseline, she could ambulate with a rolling walker without issue. She denied headaches, chest pain, dyspnea, cough, night sweats, weight loss, bowel changes, or urinary symptoms. She also denied recent travel, sick contacts, or recent illness. Her vital signs were febrile with a maximum temperature of 102F, heart rate of 104 beats per minute, blood pressure 161/72, and 94% oxygen saturation on room air. During the physical exam, she appeared to be her stated age and was comfortable in bed. Her cardiopulmonary exam showed regular S1 and S2, a systolic murmur, euvolemic status, and bilateral lung fields that were clear to auscultation. Her abdomen was soft to light and deep palpation; normoactive bowel sounds were heard, and no guarding, rebound, or rigidity. A neurological assessment showed no focal deficits, bilateral lower extremity strength was 2/5, which was at baseline, and the patient was fully alert and oriented. Gait and reflexes were not assessed. Her skin exam did not reveal any open wounds or new rashes. Initial laboratory workup was significant for marked leukocytosis and elevated inflammatory markers; all other lab values were unremarkable (Table 1). Urinalysis was negative for infection. Blood cultures were collected. Chest X-ray and contrast-enhanced computed tomography (CT) of the abdomen and pelvis did not find a source of infection to attribute to her fever. The patient was admitted under the sepsis protocol and started on broad-spectrum intravenous antibiotics with piperacillin/tazobactam and vancomycin, as well as intravenous normal saline. However, she continued to have fevers despite antibiotic therapy, therefore, Infectious Disease was consulted. On the second day of admission, blood cultures grew Staphylococcus aureus sensitive to cefazolin. A transthoracic echocardiogram was negative for valvular vegetations. Her physical exam remained unremarkable, and the source of infection remained elusive. She denies central line, pacemaker, valve surgery, or joint replacement. On the fifth day of her admission, she complained of severe back pain. She described the pain as fluctuating upper then lower back pain that was aggravated by certain movements in bed. She denied having severe back pain at baseline. Physical exam was remarkable for mild upper thoracic spinal tenderness; however, there was no tenderness over the lower back, costo-vertebral, or flank areas, and there was no notable step-off, spinal masses, or fluctuance palpated. She was given oxycodone with minimal relief. Given new back pain in the setting of methicillin-susceptible Staphylococcus aureus (MSSA) bacteremia, further imaging was warranted to assess vertebral or paravertebral infection although admission CT images did not find acute abnormalities of the spine. Magnetic resonance imaging (MRI) of her entire spine revealed a complex epidural fluid collection from C3 to L4/5 consistent with abscess, cauda equina syndrome, spinal cord compression, right psoas, and bilateral erector spinae myositis, and no evidence of osteomyelitis (Figure 1). Neurosurgery was consulted and she was managed conservatively with intravenous cefazolin, as she was deemed a poor surgical candidate. Her fevers subsided and within a couple of days, she was transferred to acute rehabilitation with plans for eight weeks of cefazolin and weekly outpatient follow-up with infectious disease.

**Table 1:** Admission laboratory investigations

Laboratory Test	Lab Value	Reference Range
Sodium (mmol/L)	136	136-145
Potassium (mmol/L)	3.4	3.5-5.1
Chloride (mmol/L)	101	98-107
CO_2_ (mmol/L)	23.1	21-31
Glucose (mg/dL)	133	70-99
Blood urea nitrogen (mg/dL)	12	7-25
Creatinine (mg/dL)	0.64	0.6-1.3
Calcium (mg/dL)	10	8.6-10.3
Anion gap (mmol/L)	12	5-12
Albumin (g/dL)	3.9	3.5-5.7
Total protein (g/dL)	7.2	6.4-8.9
Alkaline phosphatase (IU/L)	90	34-104
Aspartate aminotransferase (IU/L)	20	13-39
Alanine aminotransferase (IU/L)	13	7-52
Direct bilirubin (mg/dL)	0.2	0.0-0.2
Total bilirubin (mg/dL)	1.1	0.3-1.0
Lactic acid (mmol/L)	1.2	0.6-1.4
C-reactive protein (mg/dL)	10.09	<1.0
Erythrocyte sedimentation rate (mm/hr)	99	0-30
White blood cell count (x10^3^/µl)	38.5	4.8-10.8
Neutrophil number (x10^3^/µl)	34.33	2-8
Lymphocyte number (x10^3^/µl)	1.14	0.7-5.2
Monocyte number (x10^3^/µl)	2.07	0.1-1.3
Eosinophil number (x10^3^/µl)	0.00	0.04-0.54
Basophil number (x10^3^/µl)	0.15	0.0-0.21
Immature granulocytes number (x10^3^/µl)	0.77	0.0-0.03
Red blood cell count (x10^6^/µl)	3.98	4.5-6.1
Hemoglobin (g/dL)	12.2	14-17.5
Hematocrit (%)	36.7	39-53
Platelet count (x10^3^/µl)	524	130-400
Troponin	0.06	<0.06

## Discussion

A spinal epidural abscess (SEA) is a serious medical condition characterized by the collection of infectious fluid within the epidural space of the spine. The epidural space is located between the dura mater, the outermost layer of the spinal cord, and the vertebrae. Spinal epidural abscesses are commonly caused by bacterial infections and the most common organisms involved are Staphylococcus aureus (39%), including methicillin-resistant Staphylococcus aureus (MRSA). Other bacteria, such as Streptococcus species and gram-negative bacilli, can also be responsible. Risk factors for developing a spinal epidural abscess include immunocompromised conditions such as diabetes, HIV, or immunosuppressive therapy, recent spinal procedures or injections, and intravenous drug use. It can also be caused by hematogenous or lymphatic spread of infection from other parts of the body to the spine. A SEA is more commonly seen in the thoracolumbar spine, as the epidural space is larger in this area and contains more fat, making it more prone to infections [2]. Literature shows that SEAs secondary to septic spondylitis or intervertebral discitis tend to be located anterior to the dural tube, while those due to hematogenous infections tend to be located posterior to the dural tube [3]. Also, the epidural space is a vertical sheath-like structure and an abscess that begins at one segment can expand to other segments longitudinally [4]. In our patient, the epidural abscess was located posteriorly, and the bacteremia also points towards the hematogenous spread of infection; however, the source of infection remains unknown. 

The symptoms of a SEA can vary, but common manifestations include back pain, which is often localized based on the location of the abscess, fever and chills, neurological deficits including weakness, numbness, or tingling in the extremities, and in severe cases, paralysis, saddle anesthesia if there is compression of the cauda equina nerve roots and bowel and bladder dysfunction in advanced cases. With the classical triad for the diagnosis of SEA being fever, spinal pain, and neurological dysfunction, only a few patients have all three manifestations at the time of diagnosis [5,6]. Typically, acute cases occur when the abscess is posterior to the spinal cord, whereas chronic cases occur when the abscess is anterior to the cord [1]. Our patient did not have any focal neurological signs but had severe back pain and fever.

If there is a clinical suspicion for SEA, prompt diagnosis and management are warranted. In one study, out of 10 patients with SEA in whom the diagnosis was not made on hospital admission, 4 became paraplegic and 3 died [7]. Any delay in diagnosis can cause irreversible neurological damage and lead to significant morbidity and mortality [7].

Magnetic resonance imaging (MRI) is the imaging modality of choice for diagnosing spinal epidural abscesses, as it provides detailed visualization of the spinal structures and can reveal the extent of the abscess [1,8]. The management of a spinal epidural abscess typically involves a combination of medical and surgical interventions. Broad-spectrum antibiotics are initiated empirically and adjusted based on culture results. In cases with significant neurological compromise or abscess size, surgical drainage and decompression may be necessary.

Our patient was not deemed to be a surgical candidate because of her advanced age and co-morbidities, and hence conservative medical management was chosen after shared decision-making.

## Conclusions

This case report highlights a presentation of an extensive spinal epidural abscess in a patient with no risk factors and non-specific findings. Unfortunately, many cases go undiagnosed for several days until more obvious findings, such as neurological dysfunction, appear. Therefore, early diagnosis and prompt treatment are crucial for a favorable outcome. Delayed intervention can lead to permanent neurological damage or even death. The prognosis depends on the severity of neurological deficits, the extent of the abscess, and the timing of intervention. Rehabilitation and physical therapy may be required to address residual deficits in those who recover.
